# Symptoms of depression change with olfactory function

**DOI:** 10.1038/s41598-022-09650-7

**Published:** 2022-04-05

**Authors:** Agnieszka Sabiniewicz, Leonie Hoffmann, Antje Haehner, Thomas Hummel

**Affiliations:** 1 Interdisciplinary Center “Smell & Taste”, Department of Otorhinolaryngology, TU Dresden, Dresden, Germany; 2 Institute of Psychology, University of Wrocław, Wrocław, Poland

**Keywords:** Psychology, Medical research

## Abstract

Olfactory loss is associated with symptoms of depression. The present study, conducted on a large cohort of mostly dysosmic patients, aimed to investigate whether improvement in olfactory performance would correspond with a decrease in depression severity. In 171 participants (157 dysosmic), we assessed olfactory function and severity of depression before and after an average interval of 11 months, with many patients showing improvement in olfactory function. Separate analyses were conducted for (a) the whole group of patients and (b) the group of dysosmic patients using both classic and Bayesian approaches. For odor identification, Student *t* test demonstrated that the whole sample improved consistently, especially within the group of dysosmic patients. The dysosmic group also improved in odor threshold and overall olfactory function. Pearson correlation showed that an increase in olfactory function was associated with a decrease in depression severity, particularly in dysosmic patients. To conclude, the present results indicate that symptoms of depression change with olfactory function in general and odor identification in particular.

## Introduction

Olfactory dysfunction is related to depression^1–12^. For example, Deems and colleagues^13^ demonstrated on 750 dysosmic patients variations in depression scores. A more recent study showed that about one-fourth to one-third of patients who lost their sense of smell showed depressive symptoms^14^. Also, among older adults, impairment in olfaction was related to depression^15^. Interestingly, overall healthy older adults were more likely to develop depressive symptoms 5 or 10 years after losing their sense of smell^16^. On the other hand, depressed patients exhibit impaired olfactory function, mainly for odor identification^17–21^ but also at the level of odor thresholds^10,22–24^ and, even though there is limited evidence, for odor discrimination^3,17^.

The reciprocal relationship between olfaction and depression may result from the neuroanatomical connections between regions in the brain that are involved in both processes^2,4^. Sensory areas play an essential role in affective experience^25^. Olfactory information passes through the first central-nervous relay, the olfactory bulb, and is further conducted to the primary olfactory cortex, which then projects to the amygdala, hippocampus, anterior cingulate cortex, insula, and orbitofrontal cortex^2,26^. These areas are also involved in many affective functions such as emotional processing, as well as autonomic regulation^26^. The volume of the olfactory bulb, a highly plastic structure that is connected to the amygdala, was demonstrated to be reduced in the case of depressed patients^23^ and can be generally treated as a biological vulnerability factor for the occurrence and/or maintenance of depression^8^. Likewise, both the amygdala, a structure responsible for processing aversive stimuli and detection of emotional signals^26^ and the hippocampus, a neuroplastic temporal lobe structure responsible for both consolidating and providing an emotional context to memories^26^, are identified as primary neurobiological structures involved in the pathophysiology of depression^27–29^.

Recent studies demonstrated that mood benefits from improving olfaction via regular olfactory exposure, so-called “olfactory training” (OT), leading to a decreased severity of depressive symptoms^30,31^. The question remains open as to whether this effect is caused by the positive impact of exposure to odors on mood^32–48^ during OT or whether improvement in olfaction, even without OT, is directly related to the improvement in mood, possibly via neuroanatomical changes. Thus, in the present study, conducted on a large cohort of mostly dysosmic patients, we aimed to investigate whether improvement in olfactory performance would correspond with a decrease in depression severity and if so, to which degree. It is worth mentioning that the reasons for the dysosmia are large; from post-traumatic choc to neurodegenerative diseases and the treatments are medical. We hypothesized that improvement in olfactory function and especially in odor identification—because it has been shown to be strongly affected in depression^17,19^—is directly related to a decrease in depression severity.

## Materials and methods

### Participants

A total of 171 participants aged from 14 to 87 years of age took part in the study. Their detailed characteristics are presented in Table 1. Please, note that 44% of participants were 60 years old or older.

**Table 1:** Descriptive characteristics of the participants.

	Total
Number of participants	171
Age (M ± SD)	57.1 ± 14
Men	45
Cause of olfactory disorder	Idiopathic: 34%; postviral: 33%; sinunasal: 15%; posttraumatic: 12%; toxic: 1%; radio-chemo: 2%; postsurgery: 1%; neurodegenerative: 1%
Additional characteristics of participants potentially related to their olfactory function	Skull surgery: 2%; sinunasal surgery: 11%; polypectomy: 5%; tonsillectomy: 2%; septoplasty: 7%; nasal polyposis: 4%; Obstructive Sleep Apnea Syndrome: 2%; bronchial asthma: 8%; allergic rhinitis: 20%; chronic rhinosinusitis: 11%
Parkinson disease family history %	3%
Alzheimer disease family history %	3%
Medical treatment	Olfactory training: 80%; vitamin A nasal drops: 53%; mometasone nasal spray: 28%; roral prednisolone: 5%; azelastine plus fluticasone nasal spray: 1%; nasal douche: 5%; oral zinc gluconate: 10%; acupuncture: 5%; oral alpha lipoic acid: 2%; nasal sodium citrate spray: 1%

All participants visited the Smell and Taste Clinic of the Department of Otorhinolaryngology at the TU Dresden because of an olfactory disorder. The duration of treatment varied from 1 up to 16 months (10.9 ± 3.2) and consisted of different medical strategies presented in Table 1. Different etiologies of olfactory loss were present (Table 1).

The retrospective study was performed according to the principles of the Declaration of Helsinki on biomedical research involving human subjects. It was approved by the Ethics Committee at the Medical Faculty of the TU Dresden (EK number 251112006) covering anonymized retrospective and pooled analyses. All participants consented to the examinations.

### Olfactory testing

Detailed results of olfactory testing are presented in Table 2. The testing was conducted twice, during the first and the second visit to the Clinic. 39–40 patients did not attend visit 2—they were followed up instead with a telephone consultation.

**Table 2:** Results of Sniffin’ Sticks testing conducted during the first (before) and the second (after) visit to the Clinic.

	Time	n	Mean	SD
Threshold	Before	170	2.84	2.56
	After	131	3.27	2.55
Identification	Before	171	8.23	3.79
	After	130	9.3	3.77
Discrimination	Before	170	8.44	3.27
	After	130	9.15	3.2
TDI	Before	171	19.45	8.1
	After	132	21.42	8.55

Olfactory testing was performed by means of "Sniffin' Sticks”^49,50^ and consisted of tests for odor threshold (rose-like odor, phenylethylalcohol; PEA), odor discrimination, and odor identification. Results of the 3 subtests were presented both separately for threshold (T), with a range between 1 and 16, discrimination (D), with a range between 0 and 16 and identification (I) score, with a range between 0 and 16, and finally as a sum of the results (TDI), with the final score ranging between 1 and 48 points^50^. If the TDI score was 31 or higher, the patient was regarded as normosmic (n = 14); with a score below 31, the patient was regarded as dysosmic (n = 157)^50^.

### Neuropsychological testing

**Figure 1:**
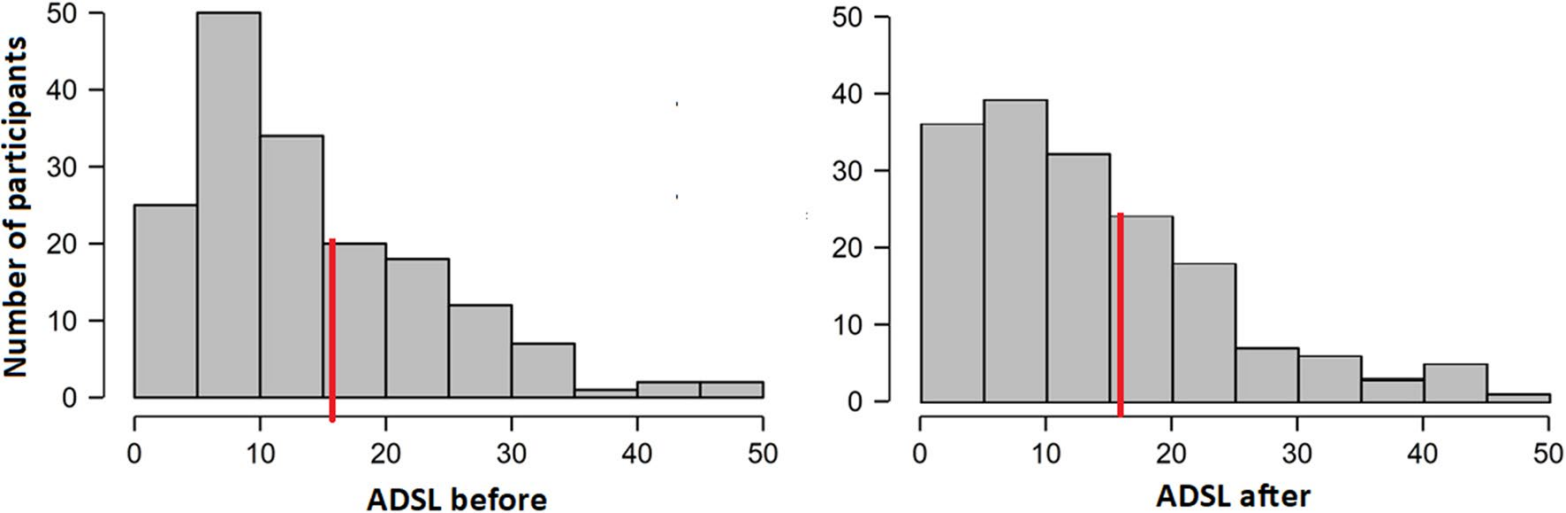
Density of results in ADSL scale in the first (before) and the second (after) measure. The red line indicates a turning point for depression^38^.

Detailed results of the depression scale are presented in Fig. 1. Like olfactory testing, depression testing was conducted twice, during the first (M = 14.32, SD = 9.89) and the second (M = 14.36, SD = 10.45) visit to the Clinic. In case of patients who were not able to come for the second visit, the measure was conducted via phone.

The General Depression Scale—long form^51,52^ (“Allgemeine Depressionsskala”—Langform; ADSL) was employed to gauge the severity of depression. The scale is a self-assessment tool that consists of 20 questions about emotional, motivational, cognitive, somatic and motor/interactional complaints, such as, e.g. ‘During the last week I hardly had an appetite’; ‘During the last week I felt as good as others’; ‘During the last week I was scared’. Due to its time-saving and cost-effective applicability, the ADSL is a practical method^51^. Patients are considered depressed when scoring 16 or more^52^. According to this cutoff point, 62 participants (36%) of the present sample were considered depressed.

### Statistics

Separate analyses were conducted for (a) the whole group of patients, with the specification of normosmic patients and (b) the group of dysosmic patients.

Possible differences between the first and the second measure in (a) olfaction and (b) depression were investigated by means of paired samples two-way *t* test. Additionally, Repeated Measures Anova was conducted to assess possible differences between dysosmic and normosmic patients, with session (before–after) as within factor and olfactory groups (normosmic vs. dysosmic) as between-subject factor. Independent samples *t* test was used to examine possible differences in depression scores between the normosmic and the dysosmic patients.

Pearson correlation was employed to investigate the relationship between the improvement in olfactory function and depression severity. Improvement in odor function was measured so that the first olfactory measure, separate for T, D, I and TDI was subtracted from the second olfactory measure. Improvement in depression was calculated in reverse: the second depression score was subtracted from the first one. This was done so that positive differences indicate improvement in olfactory function ion and depressive symptoms, respectively. Additionally, to confirm the obtained results, Principal Component Analyses was conducted (see Supplementary materials).

All the analyses were conducted twice, firstly with classical p-values and secondly with Bayesian statistics^53^. The Bayes Factor (B) is a method that weighs evidence and shows which of two hypotheses is better supported and to what extent. Adopting the B in statistical inference, it can be examined whether the data provided robust support for the null hypothesis, the alternative hypothesis, or whether the analysis is inconclusive and more data need to be collected to provide clear evidence^54^. Furthermore, Bayesian statistics are resistant to multiple comparisons.

Data are presented as mean values (± standard deviation). Statistical analyses were performed using JASP v. 0.11.1 (Univ. of Amsterdam, The Netherlands: www.jasp-stats.org), with *p* < 0.05 set as the level of significance. The effect sizes are accompanied by their 95% confidence intervals.

## Results

### Olfactory tests and depression outcome

#### The whole group of patients and normosmic patients

**Figure 2:**
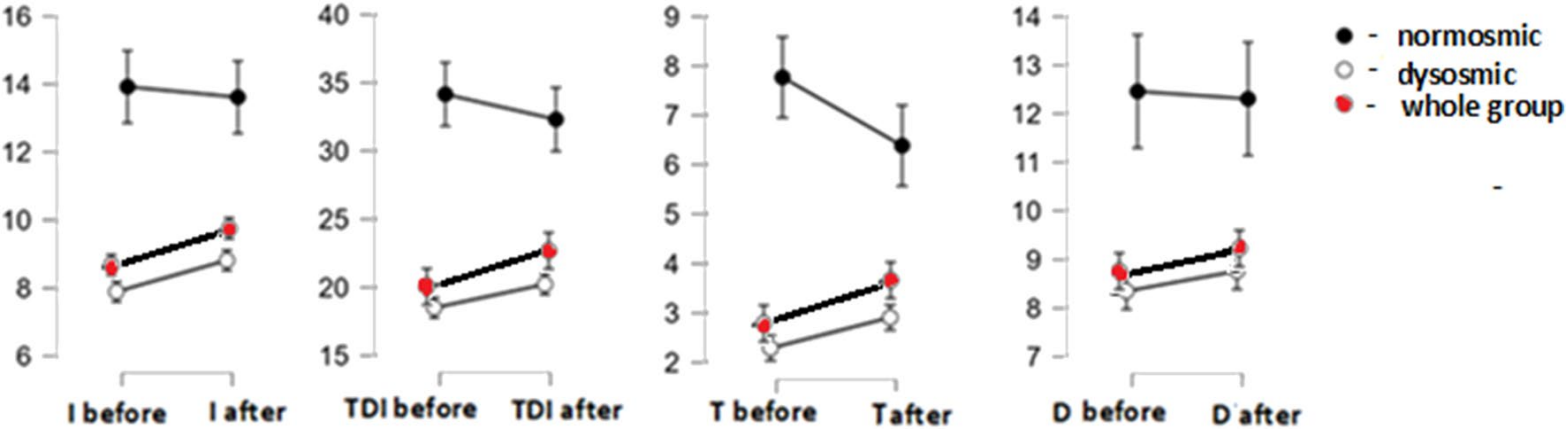
Improvement in general olfactory function (TDI), odor threshold (T), odor identification (I), and odor discrimination (D) score in the whole group of patients (marked with red color) and in the groups of normosmic and dysosmic patients (means, standard errors of means). Please, note different sizes of Y-axes.

For the whole group of patients, the classical *t* test indicated that the patients improved in terms of almost all olfactory components: odor threshold (T: t = 2.35, *p* = 0.021, Cohen’s d = 0.21), odor identification (I: t = 3.9, *p* < 0.001, Cohen’s d = 0.34) and general olfactory function (TDI: t = 2.7, *p* = 0.008, Cohen’s d = 0.24), while in the case of odor discrimination no change was noticed (D: t = 1.42, *p* = 0.16, Cohen’s d = 0.13) (Fig. 2). Normosmic patients, in turn, improved in terms of odor threshold (T: t = 2.6, *p* = 0.023, Cohen’s d = 0.72), while no change was observed in odor identification (I: t = 1.2, *p* = 0.247, Cohen’s d = 0.34), odor discrimination (D: t = 0.2, *p* = 0.843, Cohen’s d = 0.06) and general olfactory function (TDI: t = 1.2, *p* = 0.247, Cohen’s d = 0.337). The Bayesian *t* test indicated that the olfactory function did not change in normosmic patients, not even in odor threshold (for T: B10 = 2.96, for D: B10 = 0.28, for I: B10 = 0.3, for TDI: B10 = 0.51).

**Figure 3:**
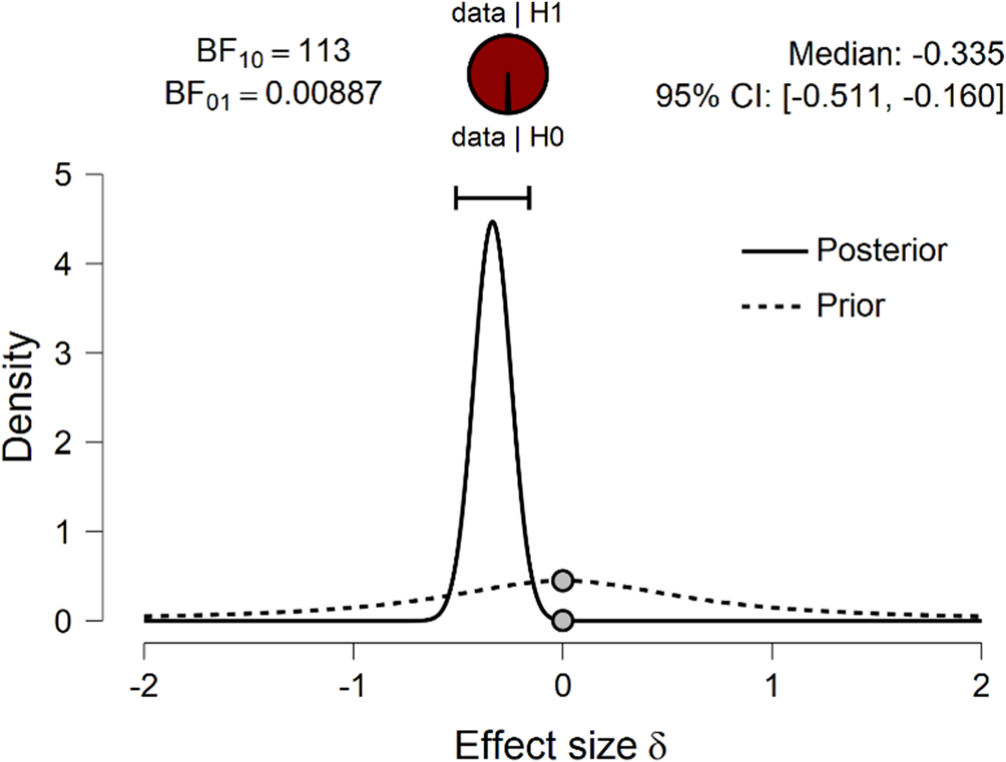
Two tailed *t* test results for odor identification (I) change in the whole group. The probability wheel on top demonstrates the evidence that the data provide for the two rival hypotheses. The two gray dots specify the prior and posterior density at the test value. The median and the 95% central credible interval of the posterior distribution are presented in the top right corner^71^.

The Bayesian *t* test for the whole group of patients demonstrated slightly different results (Fig. 3). While the Bayes factor supported very strongly the presumption about a change in odor identification performance (I: B10 = 112.77), neither odor threshold (T: B10 = 1.36) nor discrimination (D: B10 = 0.26) was shown to change, and in these cases the Bayes factor supported H0. Instead, general olfactory function changed slightly (TDI: B10 = 3.1), which was probably driven by the large change in odor identification.

In terms of the depression score the classical *t* test indicated no evidence supporting the difference between the first (for the whole group: M = 14.3, SD = 9.9; for the normosmic patients: 18.1, SD = 11) and the second measure in depression (for the whole group: M = 14.4, SD = 10.4; for the normosmic patients: M = 15.4, SD = 11.8) (for the whole group: t = 0.07, *p* = 0.94, Cohen’s d = 0.005; for the normosmic patients: t = 1.3, *p* = 0.21, Cohen’s d = 0.35). The Bayesian *t* test results provided further strong evidence for the null hypothesis stating that both measures did not, indeed, differ from each other (B01 = 11.69).

#### Dysosmic patients

For the group of dysosmic patients, the classical *t* test indicated that the dysosmic patients improved in terms of three out of four olfactory components: odor threshold (T: t = 3.42, *p* < 0.001, Cohen’s d = 0.32), identification (I: t = 4.35, *p* < 0.001, Cohen’s d = 0.4) and general olfactory function (TDI: t = 3.24, *p* = 0.002, Cohen’s d = 0.3), while odor discrimination value did not change (D: t = 1.55, *p* = 0.12, Cohen’s d = 0.12) (Fig. 2). Dysosmic patients had lower olfactory function in all investigated aspects compared to normosmic patients (for T: F[1,128] = 66.7, *p* < 0.001, η^2^ = 0.29; for D: F[1,127] = 25, *p* < 0.001, η^2^ = 0.13; for I: F[1,128] = 32.6, *p* < 0.001, η^2^ = 0.18; for TDI: F[1,130] = 53, *p* < 0.001, η^2^ = 0.25) (see: Fig. 2).

The Bayesian *t* test further demonstrated that the hypotheses about change in odor identification, threshold and general olfactory function were strongly supported (for I: B10 = 535.09, for T: B10 = 23.505, for TDI: B10 = 14.02). At the same time, analysis of odor discrimination data indicated the null hypothesis (D: B10 = 0.329).

Like in the case of the whole group, depression severity did not change significantly (for the first measure: M = 14, SD = 9.8; for the second measure: M = 14.3, SD = 10.4), which was indicated by the classic *t* test (t = 0.47, *p* = 0.64, Cohen’s d = 0.04). The Bayesian test, additionally, provided strong evidence for the lack of a difference between the first and the second measure (BF01 = 10.11).

Furthermore, no difference on the assumed significance level was found between the dysosmic and normosmic group in terms of depression severity, either for the first measure (but see size effect: t = 1.49, *p* = 0.14, Cohen’s d = 0.42) or the second one (t = 0.4, *p* = 0.69, Cohen’s d = 0.11). The Bayesian factor indicated that the severity of depression in the second measure was the same in both groups while in the first measure more data would be needed to support or reject this hypothesis (B01 for the first measure = 1.44; for the second measure = 3.35).

### Pearson correlations

#### The whole group of patients

For the whole group of patients, no correlation was observed between the change in depression severity and the change in odor discrimination (r = 0.09, *p* = 0.33), and odor threshold (r = 0.17, *p* = 0.84). Instead, change in depression severity correlated positively with the change in general olfactory function (TDI: r = 0.25, *p* = 0.004) and in odor identification (I: r = 0.22, *p* = 0.011). This finding was, respectively, strongly and moderately confirmed by the Bayesian factor (for “TDI score” B10 = 12.8, for I score: 5.51 (Supplemental Table 1).

### Dysosmic patients

**Figure 4:**
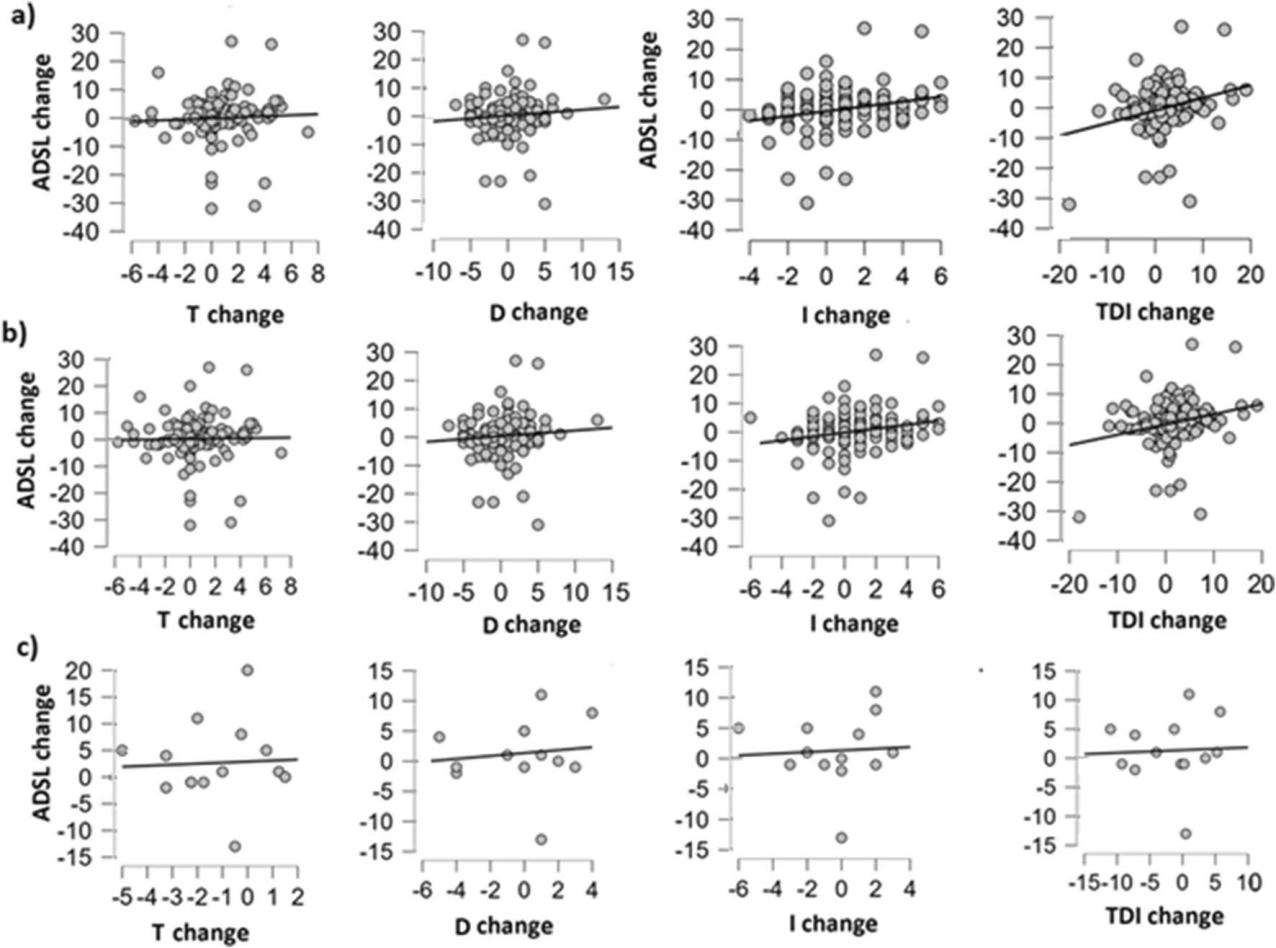
Classical Pearson one-way correlation between, respectively, the change in T, D, I and TDI score and depression severity (ADSL) score for (**a**) the group of dysosmic patients; (**b**) the whole group; (**c**) the group of normosmic patients.

For the group of dysosmic patients, no correlation was observed between the change in depression severity and the change in odor discrimination (r = 0.09, *p* = 0.35), or odor thresholds (r = 0.05, *p* = 0.60). Instead, changes in depression severity correlated positively with the change in general olfactory function (r = 0.28, *p* < 0.001) and in odor identification (r = 0.25, *p* = 0.003) (Fig. 4). This was confirmed by Bayesian statistics, strongly for general olfactory function (B10 = 28.5) and moderately for odor identification (B10 = 8.8) (Supplemental Table 2).

## Discussion

In order to explore the idea that depressive symptoms can benefit from improved olfaction via repeated exposition to odors, we examined whether olfactory improvement is associated with a reduction in depression severity. Specifically, in a large sample of primarily dysosmic patients, we sought evidence for an association between change in depression severity (measured by ADSL scale^52^) and variation in olfactory function (namely odor threshold (T), discrimination (D), identification (I), and general olfactory function (TDI) as measured by the Sniffin Sticks test battery^49,50^). In accordance with our assumption, the present results show that an increase in olfactory function corresponded moderately to strongly with the decrease in depressive symptoms, which was particularly notable in the group of dysosmic patients. Furthermore, the whole sample improved consistently, especially within the group of dysosmic patients in terms of I, and the dysosmic group also improved in terms of T and TDI.

The majority of the participants in the present study were dysosmic due to viral infections, sinonasal disease, and traumatic brain injury, which are the most typical causes of olfactory dysfunction^13,55–57^. Sinonasal disease may lead to a gradual loss of olfactory function generated by nasal obstruction blocking access of odors to the olfactory cleft and chronic inflammation of the olfactory epithelium^58^. In addition, infections have been demonstrated to result in damage to the olfactory mucosa, thereby disabling the function and regeneration of olfactory receptor neurons^59,60^. Finally, head trauma may disrupt olfactory pathways or damage olfactory brain areas^61,62^.

Previous studies demonstrated that dysosmic patients can benefit from OT to improve their sense of smell and reduce their symptoms of depression^30,31^. Odor identification is an olfactory dimension with a solid cognitive association^63^ that has been consistently reported to be impaired in depression^17–21^, probably because of the cognitive impairment in recurrent depression^19^. Specifically, reduced attention to and memory of olfactory stimuli may lead to decrease in odor identification^2^. Furthermore, odor identification is also more subject to complex processing compared to odor thresholds^63^ and thus may show more overlap with areas that are involved in the processing of emotions, and hence, depression. Bestgen and colleagues^64^ showed that odor identification was predicted by odor emotional quality, and a number of studies indicated its relation with depression^17–21^.

The present finding indicating a relationship between odor identification and depression severity in dysosmic patients may also be linked to the large improvement in odor identification in this group, which was five times greater than in the whole sample. We presume that better general olfactory and odor identification performance may lead to cognitive and affective improvement.

Notably, the majority of the sample in the present study was not clinically depressed, as indicated by the results of the ADSL scale^52^. However, according to the previously mentioned scale, a significant percentage of the present sample (one-third of the entire sample) was classified as depressed. That could probably explain why depression severity before and after medical olfactory treatment described in Table 1 remained the same, contrary to previous studies involving OT^30,31^. In this context, strong and moderate relationships obtained between change in TDI, I, and depression severity suggest that changes in olfaction can be related even to very subtle changes in mood.

The relationship between changes in olfaction and mood is also underlined by the difference between correlation results in both groups. It is worth noting that in the group of dysosmic patients, both improvement of general olfactory function (TDI) and odor identification score (I) corresponded with a decrease in depression severity to a higher degree than in the whole sample of patients which included several normosmic participants.

Olfactory impairment has been demonstrated to coexist with depression both in the general population^1,6,10^ and in specific groups, such as older adults [^15,16^,^65,66^; but see also:^67^]. This age group is particularly prone to reduced olfaction^68^. Hence, this group appears to be specifically exposed to the consequences of olfactory dysfunction. In a very recent study, Qazi and colleagues^66^ demonstrated that olfactory impairment predicted the occurrence of depressive symptoms in a group of older adults. In line with this evidence, the present study results, where almost half of the participants were older than 65 years old, show that controlling for olfactory function might be an efficient tool to mediate mood changes.

The present study is not free from limitations. Firstly, because of the outbreak of the pandemic COVID-19, 23% of patients did not participate in person in the follow-up session and were examined only by a phone interview. Secondly, it should be noted that correlation results between the change in odor identification and depression severity were moderate according to Bayesian classification. Nevertheless, depression severity is a complex issue with a number of varying factors related to it^69^, and odor identification is only one among them. Future studies in this field should further examine this issue. Also, patients who suffer emotionally from their smell impairment are more likely to visit specialized centres on smell and taste and to return for re-testing. Thus, the present study sample probably showed more depressive symptoms compared to other people with smell impairment who do not search for clinical counseling^70^.

To conclude, the present study results conducted on a large cohort of mostly dysosmic patients indicate that olfactory function in general (TDI), and odor identification (I) in particular, may be used as an indicator of subtle mood changes. Further, the results suggest an improvement of symptoms of depression with an improvement in the sense of smell.

## Supplementary Information

Supplementary Information.

## Data Availability

The datasets analyzed during the current study are not publicly available due to the privacy of the participants but are available from the corresponding author on reasonable request.
