# Supplementary material for: Symptoms of depression change with olfactory function
Source: Sci Rep. 2022 Apr 5;12:5656. doi: 10.1038/s41598-022-09650-7 (PMC8983665; doi:10.1038/s41598-022-09650-7)
Supplement: Supplementary file 1 — Supplementary Information. [file 41598_2022_9650_MOESM1_ESM.pdf]

Symptoms of depression change with olfactory function

Agnieszka Sabiniewicz*[1,2], Leonie Hoffmann[1], Antje Haehner[1], Thomas Hummel[1]

1 Interdisciplinary Center „Smell & Taste“, Department of Otorhinolaryngology, TU Dresden, Dresden, Germany;

2 Institute of Psychology, University of Wrocław, Wrocław, Poland;

* corresponding author. e-mail address: a.sabiniewicz@gmail.com, ORCID number: 0000-0002-9361-0486

Supplemental Table 1

Bayesian Pearson one-way correlation between the change in olfactory function and depression severity (ADSL) score for the whole group of patients

		r	BF_{+0}
change in ADSL	TDI change	.25	12.798
change in ADSL	I change	.22	5.509
change in ADSL	D change	.09	0.287
change in ADSL	T change	.17	0.128

Supplemental Table 2

Bayesian Pearson one-way correlation between the change in olfactory function and depression severity (ADSL) score for the group of dysosmic patients

		r	BF_{+0}
change in ADSL	TDI change	.24	15.339
change in ADSL	I change	.19	3.030
change in ADSL	D change	.08	0.275
change in ADSL	T change	.05	0.183

Statistics

Principal Component Analyses with oblique rotation was performed for ADSL change and changes in all measured olfactory functions (T, D, I, and TDI), separately for the whole group and the group of dysosmic patients. Component loading shows individual variable loadings on the emerged components. Uniqueness presents the percentage of the variance of each variable that is not explained by the component. The results were further

illustrated by a) path diagrams (Figure 1 and 3) that display visual representation of the direction and strength of the relation between the variable and component and b) scree plots (Figure 2 and 4) that provide information on how much variance in the data, indicated by the eigenvalue, is explained by each component.

Results

The whole group of patients

Two main components emerged, the first one (RC1) consisted of TDI change (component loading = .94, uniqueness = .013), D change (component loading = .796, uniqueness = .398), T change (component loading = .764, uniqueness = .446), and I change (component loading = .429, uniqueness = .424). The second one (RC2) consisted of I change (component loading = .536, uniqueness = .424) and change in ADSL (component loading = .9, , uniqueness = .215) (Figure 1).

Figure 1 Visual representation of the direction and strength of the relation between the variables and components in the whole group

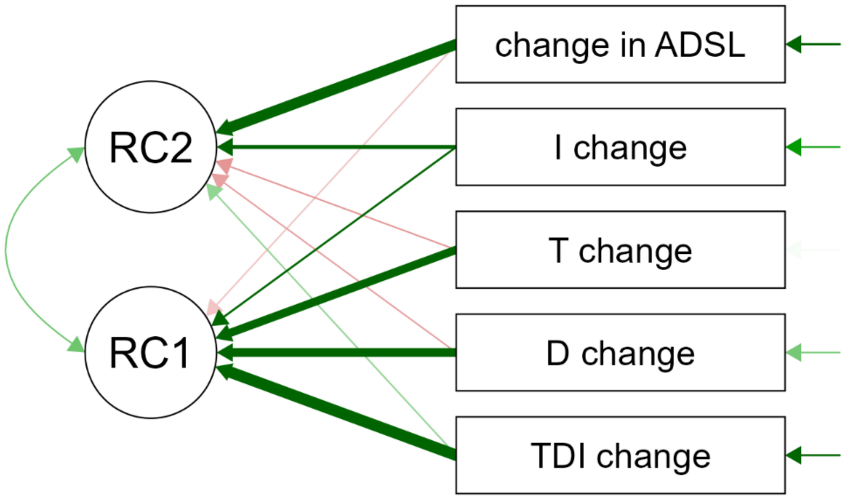

RC1 explained 4.9% and RC2 explained 2.1% of the data variance (for RC1: proportion variance = .492; for RC2: proportion variance = .208). Both components eigenvalue was above 1 (for RC1: eigenvalue = 2.46, for RC2: eigenvalue = 1.04), what is presented on Figure 2.

Figure 2 Eigenvalue of RC1 and RC2 in the whole group

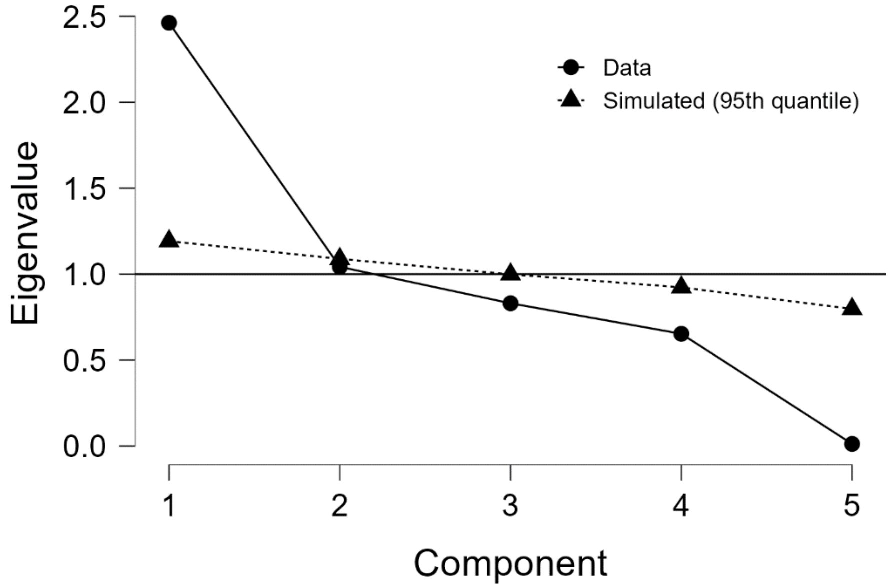

Dysosmic patients

Like in the whole group of patients, here two main components emerged. The first one (RC1) consisted of TDI change (component loading = .91, uniqueness = .013), D change (component loading = .837, uniqueness = .336), and T change (component loading = .72, uniqueness = .506). The second one (RC2) consisted of I change (component loading = .662, uniqueness = .383) and change in ADSL (component loading = .856, , uniqueness = .295). Input of individual components is presented on the Figure 3.

Figure 3 Visual representation of the direction and strength of the relation between the variables and components in dysosmic patients

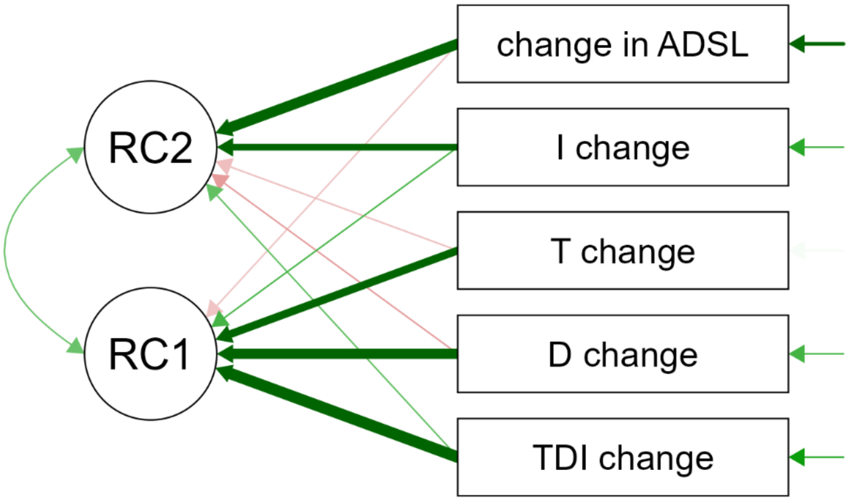

RC1 explained 4.8% and RC2 explained 2.1% of the data variance (for RC1: proportion variance = .483; for RC2: proportion variance = .211). Both components eigenvalue was above 1 (for RC1: eigenvalue = 2.4, for RC2: eigenvalue = 1.05), what is presented on Figure 4.

Figure 4 Eigenvalue of RC1 and RC2 in dysosmic patients

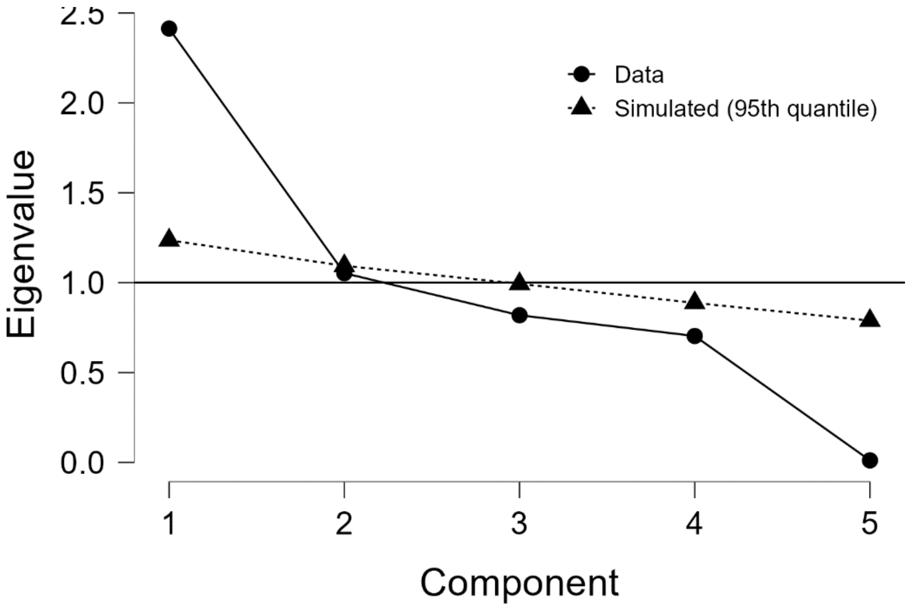

Present results indicate a relationship between change in depression severity and change in odor identification. Please note that in both groups, change in odor identification was the only one significantly related to change in depression severity. This correlation was even more visible in dysosmic patients, thereby confirming our previous findings.
